# Analysis of risk factors for recurrence after laparoscopic myomectomy: A retrospective study

**DOI:** 10.1097/MD.0000000000041697

**Published:** 2025-03-14

**Authors:** Tingzhu Meng, Shiyu Cheng, Xin Li, Han Gao, Yanli Li, Mei Du, Jie Shi

**Affiliations:** a Department of Gynecology, Maternal and Child Health Hospital of Hubei Province, Tongji Medical College, Huazhong University of Science and Technology, Wuhan, China; b Medical College, Wuhan University of Science and Technology, Wuhan, China.

**Keywords:** laparoscopic myomectomy, postoperative recurrence, prognosis, risk factors, uterine leiomyoma

## Abstract

At present, there is unclear on the risk factors of recurrence after myomectomy. In this study, we hope to provide reference for the choice of treatment for patients with uterine fibroids and provide basis for the subsequent prediction of recurrence. From October 2020 to October 2022, we enrolled 240 patients with uterine fibroids in Hubei Maternal and Child Health Hospital. According to the inclusion criteria, the collected clinical data of these patients were analyzed and divided into 2 groups according to whether there was recurrence 6 months after surgery(a recurrence group [52 cases] and a non-recurrence group [78 cases]). We compared and analyzed the relevant factors. Univariate analysis showed that there was no significant relationship between fibroid diameter, postoperative pregnancy, contraceptive method, hyperlipidemia, diabetes, endometriosis and postoperative recurrence (*P* > .05). While, age, number of pregnancies, number of fibroids, type of fibroids, body mass index, endometrial hyperplasia or endometrial polyp were significantly correlated with postoperative recurrence (*P* < .05). Multivariate Logistic regression analysis demonstrated that body mass index > 24, number of pregnancies > 2, multiple myoma, intermyoma and endometrial hyperplasia were independent risk factors for postoperative myoma recurrence (*P* < .05). Body mass index > 24, number of pregnancies > 2, multiple fibroids, intermyowall fibroids, and endometrial hyperplasia are all independent risk factors for recurrence after laparoscopic myomectomy in patients with uterine fibroids. Patients with these independent risk factors should be closely reviewed. If they have multiple independent risk factors and have no fertility requirements are present, hysterectomy may be recommended.

## 1. Introduction

Uterine leiomyoma is the most common benign gynecological tumor, which originates from the clonal expansion of individual cells in the myometrium.^[1]^ As uterine leiomyoma are affected by hormones, uterine fibroids mainly affect women of childbearing age, with a prevalence of up to 70%.^[2]^ The vast majority of patients have no obvious clinical symptoms, most of which are found through imaging examination. Only about 30% of patients have relatively serious clinical symptoms,^[3]^ such as abnormal uterine bleeding, severe anemia, constipation, low back pain, and frequent urination. In severe cases, pregnancy outcome may even be affected, leading to abortion, premature delivery, or even fetal death.^[4]^ For uterine fibroids, the current surgical methods are broadly categorized into hysterectomy or myomectomy. Gynecologists usually choose Laparotomy or laparoscopic surgery to perform these operations. A recent study found that laparoscopic surgery can reduce intraoperative blood loss, shorter the overall hospital stays, and reduce postoperative pain, there is no difference between the 2 for patients’ postoperative recovery status and pregnancy outcome.^[5]^ So with the continuous development of medical level, most women will choose minimally invasive hysteromyoma resection,^[6]^ but it is easy to relapse after surgery. The money, time and clinical symptoms generated by the disease bring a double burden on patients both physically and psychologically.^[7]^ At present, there is unknown on the risk factors for fibroid recurrence, and a large number of studies are still needed for analysis. This study aims to explore the risk factors of recurrence after laparoscopic myomectomy, and provide certain basis for postoperative prevention and treatment of patients.

## 2. Materials and methods

### 2.1. Research object

From October 2020 to October 2022, we recruited 240 patients with uterine fibroids in Hubei Maternal and Child Health Hospital. Inclusion criteria: age between 2 0 and 50 years; laparoscopic myomectomy was performed in our hospital and confirmed by postoperative examination; myomectomy had not been performed before; regular postoperative review. Exclusion criteria: imaging examination indicated residual myoma lesions after surgery; complicated with cervical and endometrial malignant lesions; patients undergoing hysteroscopic myomectomy at the same time; history of myomectomy. According to the above criteria, 130 patients were finally included in the study, and were divided into a recurrence group (52 cases) and a non-recurrence group (78 cases) according to whether they relapsed 6 months after surgery. The project was approved by the Ethics Committee of Maternal and Child Health Hospital of Hubei Province (No. [2023] IEC (A138)).

### 2.2. Research data

Data were collected and analyzed, which included age, body mass index, comorbidities (hyperlipidemia, diabetes, endometrial hyperplasia, endometrial polyps, and endometriosis), number of pregnancies, number of uterine fibroids, maximum diameter of uterine fibroids, type of fibroids, postoperative pregnancy, and contraceptive method.

### 2.3. Postoperative follow-up

Patients were followed up through telephone and outpatient electronic data retrieval, and the follow-up was up to October 2023. The criteria for postoperative recurrence were as follows: the ultrasound was normal at 3 months postoperatively, but at 6 months postoperatively it suggested a leiomyosarcoma which was judged to be recurrent.^[8]^

### 2.4. Statistical method

SPSS 26.0 statistical software was used to calculate and analyze the data, and the count data were expressed as (cases [%]) by χ^2^ test; the risk factors were analyzed by multifactorial logistic regression analysis. The difference was considered statistically significant at *P* < .05.

## 3. Results

We recruited 240 patients, 98 patients failed the inclusion criteria. The remaining 142 patients were followed up, among which 11 patients could not be contacted due to the change of phone number. The final sample size in this study was 130 patients.

In this study, the total number of relapses was 52, and the overall relapse rate was 40%. We divided the age group at the node of 45 years by whether or not there was a request for childbearing, and we found significantly higher recurrence rates in patients younger than 45 years of age. Based on the patient’s general condition, which will be analyzed by body mass index, number of pregnancies, whether or not the pregnancy was postoperative, and whether or not there was a combination of hyperlipidemia and diabetes. The recurrence rate of patients in the obese group was 53%, and the recurrence rate of patients with more than 2 pregnancies was higher than that of patients with a single pregnancy, while diabetes, hyperlipidemia and postoperative pregnancy had no correlation with leiomyosarcoma recurrence. Among the basic characteristics of leiomyosarcomas, the recurrence rate of intermural leiomyosarcomas was 58% and that of multiple leiomyosarcomas was 67%, both of which correlated with leiomyosarcoma recurrence. Endometrial polyps, endometrial hyperplasia and endometriosis correlate with sex hormone levels, and some patients with fibroids are susceptible to a combination of endometrial polyps, endometrial hyperplasia or endometriosis. Therefore, these 3 indicators were included in the analysis, and the results showed endometrial hyperplasia or endometrial polyps were correlated with the recurrence of uterine fibroids. Above all, there was no significant relationship between fibroid diameter, postoperative pregnancy, contraceptive method, hyperlipidemia, diabetes, endometriosis and postoperative recurrence (*P* > .05). However, age <45 years old, pregnancy frequency more than 2 times, multiple fibroids, intermuscular fibroids, obesity, endometrial hyperplasia or endometrial polyps are the high risk factors for postoperative recurrence in patients(*P* < .05; Table 1).

**Table 1:** Univariate analysis of recurrence after laparoscopic myomectomy.

Observation target	Non-recurrence group (78 cases)	Recurrence group (52 cases)	χ2	*P*
Age (cases)			3.883	<.05
≦45	60	47		
>45	18	5		
Number of pregnancies			12.897	<.05
≤2	52	18		
>2	26	34		
Number of uterine fibroids			11.662	<.05
1	55	21		
≥2	23	31		
Maximum diameter of uterine fibroids			0.227	>.05
≤5	21	16		
>5	57	36		
Type of fibroids			16.508	<.05
Subserous myoma	65	26		
Intramural myoma	13	26		
Postoperative pregnancy			0.000	>.05
No	71	48		
Yes	7	4		
BMI			4.871	<.05
>24	20	23		
≦24	58	29		
Contraceptive method			2.229	>.05
Condom	40	20		
Contraceptive	16	15		
Intrauterine ring	22	17		
Endometrial hyperplasia			9.921	<.05
Yes	24	29		
No	54	23		
Endometrial polyps			5.098	<.05
Yes	21	24		
No	57	28		
Endometriosis			0.067	>.05
Yes	18	11		
No	60	41		
Hyperlipidemia or diabetes			0.362	>.05
Yes	16	13		
No	62	39		

BMI = body mass index.

In the multivariate logistic regression analysis, recurrence after laparoscopic myomectomy was taken as the dependent variable, and the risk factors with statistical significance in the univariate analysis were taken as independent variables. The results demonstrated that body mass index > 24, number of pregnancies > 2, multiple fibroids, intermuscular fibroids and endometrial hyperplasia were all independent risk factors for recurrence after laparoscopic myomectomy (OR = 6.916, 7.017, 4.114, 3.872, 3.578), and the differences were statistically significant (all *P* < .05; Table 2).

**Table 2:** Multivariate logistic regression analysis of recurrence after laparoscopic myomectomy.

Variable	β	SE	Wald χ2	*P*	OR (95%CI)
Age	−1.125	0.726	2.401	>.05	0.325 (0.078, 1.347)
Number of pregnancies	1.948	0.517	14.219	<.05	7.017 (2.549, 19.318)
Number of uterine fibroids	1.414	0.496	8.139	<.05	4.114 (1.557, 10.871)
Type of fibroids	1.354	0.524	6.685	<.05	3.872 (1.388, 10.805)
BMI	1.934	0.561	11.866	<.05	6.916 (2.301, 20.783)
Endometrial hyperplasia	1.275	0.535	5.681	<.05	3.578 (1.254, 10.207)
Endometrial polyps	0.942	0.515	3.342	>.05	2.564 (0.934, 7.037)

BMI = body mass index, CI = confidence interval, OR = odds ratio, SE = standard error.

## 4. Discussion

As the incidence of uterine fibroids is increasing year by year, it has become a gynecological disease of greater concern to women. The majority of uterine fibroids are benign lesions, and the probability of malignant transformation in the world is <1%.^[9]^ In clinical treatment of uterine fibroids, most of the surgical methods are adopted, the rate of recurrence of leiomyosarcoma varies from 10% to 40%. The risk of postoperative recurrence is a difficult problem we are currently facing. Glover et al^[10]^ conducted a survey of 200 women with uterine fibroids in London and found that 35% of women had depression scores in the critical or clinical range and 61% had anxiety scores in the critical or clinical range. However, patients with uterine fibroids not only suffer from depression and anxiety, but also have a higher incidence of self-directed violence and stress.^[11]^ These literatures all indicate that uterine fibroids have a serious impact on the quality of life of women, so it is urgent to reduce postoperative recurrence, carry out targeted treatment for relapse-prone groups and improve prognosis. Although there is a large amount of literature on postoperative recurrence of uterine fibroids, there is still a lack of uniform consensus, so we carried out this study and determine to provide reference for the prevention of postoperative recurrence of patients with uterine fibroids.

In this study, multiple fibroids, intermyowall fibroids, body mass index > 24, number of pregnancies > 2 and endometrial hyperplasia were all independent risk factors for recurrence after laparoscopic myomectomy in patients with uterine fibroids. To analyze the reasons why multiple leiomyomas and intermural leiomyomas are prone to recurrence, there may be the following 2 points. First, all current surgical methods require the surgeon to assist with imaging reports during the operation and perform visual exploration of fibroids. However, excessive number of fibroids and their location in the deeper layers of the muscle make removal of fibroids more difficult. Second, the pathogenesis of uterine fibroids is still unclear.^[12]^ Patients may have mutagenic factors of fibroids in their bodies, and the more fibroids, the more such mutagenic factors, resulting in a higher postoperative recurrence rate of patients. At present, there is no definite evidence of the developmental relationship between obesity and uterine leiomyoma. However, some scholars^[13]^ believe that in obese women, hormones secreted by adipocytes or adipocytes may promote the development and growth of leiomyoma by activating leptin receptor signaling pathway. In addition, It has been reported in literature^[14]^ that leiomyoma is a monoclonal tumor in which myogenic stem cells become tumorigenic after mutation, abnormal methylation or abnormal signal transduction, and obesity may mediate myogenic stem cell to initiate tumorigenesis and lead to leiomyoma. The above reports may be the cause of the risk factors for fibroid recurrence caused by obesity. It was reported^[15]^ that excessive number of pregnancies may lead to long-term inflammatory state of the body, which will aggravate the proliferation of fusiform smooth muscle cells and fibrous connective tissue, and increase the expression of sex hormone receptors. Each pregnancy will damage the normal physiological structure of the uterus, and the recovery of the uterus will take some time. Multiple pregnancies lead to a lack of recovery time and an increased risk of recurrence of fibroids. Patients with endometrial hyperplasia have a high level of estrogen, which causes uterine fibroids to proliferate,^[16]^ so this may be the reason why patients with combined endometrial hyperplasia have an increased risk of postoperative recurrence.

It is well known that uterine leiomyomas are estrogen-dependent, with higher estrogen receptors than surrounding normal tissues.^[17]^ Increased proliferation of uterine fibroids was observed in vitro when exposed to estradiol and progesterone.^[18]^ For patients who have undergone hysteromyomectomy, strict contraception is required for a period of time. At present, all drug contraception methods contain estrogen or progesterone. According to the different accompanying symptoms of uterine fibroids, different contraceptive methods can also effectively reduce the occurrence of such symptoms.^[19]^ It had been reported^[20]^ that the use of contraceptives can reduce the occurrence of uterine fibroids in the surveyed women, especially in patients aged 30 to 40 years. Therefore, contraception is considered to be a protective factor for the development of uterine fibroids. However, some studies^[21]^ have suggested that long-term use of oral contraceptives cannot increase the risk of uterine leiomyoma in women. Other study^[22]^ believes that there is insufficient evidence to prove that contraceptive devices can reduce the size of uterine fibroids. In this study, different contraceptive methods had no statistical significance on the risk of fibroid recurrence, which was considered because the sample size was too small, resulting in a lack of reference data. Endometriosis is also a hormone-dependent disease, and Lin et al^[23]^ believed that uterine smooth muscle tumors increase the risk of endometriosis development, but there was no literature showing that endometriosis patients would increase the occurrence of uterine fibroids. Unfortunately, although this study was conducted in this paper, due to the small sample size, the data obtained were of no obvious reference value.

## 5. Conclusions

Uterine fibroids account for a very heavy proportion of gynecological diseases. The problem of recurrence of the disease has been puzzling patients and clinicians. Therefore, it is extremely important to assess the recurrence risk of patients after laparoscopic myomectomy. In the study, we found that body mass index > 24, number of pregnancies > 2, multiple leiomyomas, interstitial leiomyomas, and combined endometrial hyperplasia are high-risk independent factors for postoperative leiomyoma recurrence. We could assess the risk of recurrence in patients with these risk factors before surgery, and provide targeted treatment and follow-up to reduce the recurrence rate and improve the postoperative quality of life of female patients.

## 6. Limitations

Due to the small sample size of this study and the fact that it was only a single-center study, it could not extensively cover the overall female population and may only be of reference value to women in the region.

## Author contributions

**Conceptualization:** Tingzhu Meng, Jie Shi.

**Data curation:** Tingzhu Meng, Shiyu Cheng.

**Formal analysis:** Tingzhu Meng, Shiyu Cheng.

**Investigation:** Xin Li, Mei Du.

**Methodology:** Tingzhu Meng, Shiyu Cheng, Xin Li.

**Project administration:** Han Gao, Yanli Li.

**Validation:** Jie Shi.

**Writing – original draft:** Tingzhu Meng.

**Writing – review & editing:** Jie Shi.

## References

[1] Machado-Lopez A Simón C Mas A. Molecular and cellular insights into the development of uterine fibroids. Int J Mol Sci. 2021;22:8483. 34445194 10.3390/ijms22168483 PMC8395213

[2] Stewart EA Cookson CL Gandolfo RA Schulze-Rath R. Epidemiology of uterine fibroids: a systematic review. BJOG. 2017;124:1501–12. 28296146 10.1111/1471-0528.14640

[3] Giuliani E As-Sanie S Marsh EE. Epidemiology and management of uterine fibroids. Int J Gynaecol Obstet. 2020;149:3–9. 31960950 10.1002/ijgo.13102

[4] Coutinho LM Assis WA Spagnuolo-Souza A Reis FM. Uterine fibroids and pregnancy: how do they affect each other? Reprod Sci. 2022;29:2145–51. 34142343 10.1007/s43032-021-00656-6

[5] Giannini A Cuccu I D’Auge TG. The great debate: surgical outcomes of laparoscopic versus laparotomic myomectomy. A meta-analysis to critically evaluate current evidence and look over the horizon. Eur J Obstet Gynecol Reprod Biol. 2024;297:50–8. 38581885 10.1016/j.ejogrb.2024.03.045

[6] Qu Y Chen L Guo S Liu Y Wu H. Genetic liability to multiple factors and uterine leiomyoma risk: a Mendelian randomization study. Front Endocrinol (Lausanne). 2023;14:1133260. 37576957 10.3389/fendo.2023.1133260 PMC10415162

[7] Ghant MS Sengoba KS Recht H Cameron KA Lawson AK Marsh EE. Beyond the physical: a qualitative assessment of the burden of symptomatic uterine fibroids on women’s emotional and psychosocial health. J Psychosom Res. 2015;78:499–503. 25725565 10.1016/j.jpsychores.2014.12.016

[8] Han H Han W Su T Shang C Shi J. Analysis of risk factors for postoperative bleeding and recurrence after laparoscopic myomectomy in patients with uterine fibroids: a retrospective cohort study. Gland Surg. 2023;12:474–86. 37200927 10.21037/gs-23-92 PMC10186162

[9] Radosa MP Owsianowski Z Mothes A. Long-term risk of fibroid recurrence after laparoscopic myomectomy. Eur J Obstet Gynecol Reprod Biol. 2014;180:35–9. 25016181 10.1016/j.ejogrb.2014.05.029

[10] Glover L Novakovic A Hunter MS. An exploration of the nature and causes of distress in women attending gynecology outpatient clinics. J Psychosom Obstet Gynaecol. 2002;23:237–48. 12520861 10.3109/01674820209074678

[11] Chiuve SE Huisingh C Petruski-Ivleva N Owens C Kuohung W Wise LA. Uterine fibroids and incidence of depression, anxiety and self-directed violence: a cohort study. J Epidemiol Community Health. 2022;76:92–9. 34301795 10.1136/jech-2020-214565 PMC8666805

[12] Alset D Pokudina IO Butenko EV Shkurat TP. The effect of estrogen-related genetic variants on the development of uterine leiomyoma: meta-analysis. Reprod Sci. 2022;29:1921–9. 35414045 10.1007/s43032-022-00911-4

[13] Afrin S Ramaiyer M Begum UAM Borahay MA. Adipocyte and adipokines promote a uterine leiomyoma friendly microenvironment. Nutrients. 2023;15:715. 36771423 10.3390/nu15030715 PMC9919329

[14] Afrin S Kirschen GW Borahay MA. Niche to leiomyoma via inducing oxidative stress, DNA damage, proliferation, and extracellular matrix deposition. Genes (Basel). 2023;14:1625. 37628676 10.3390/genes14081625 PMC10454202

[15] Wang H Li C Chen L Zhang M Ren T Zhang S. Causal relationship between female reproductive factors, sex hormones and uterine leiomyoma: a Mendelian randomization study. Reprod Biomed Online. 2024;48:103584. 38061975 10.1016/j.rbmo.2023.103584

[16] Mavrelos D Ben-Nagi J Holland T Hoo W Naftalin J Jurkovic D. The natural history of fibroids. Ultrasound Obstet Gynecol. 2010;35:238–42. 20069541 10.1002/uog.7482

[17] Grings AO Lora V Ferreira GD Brum IS Corleta Hv Capp E. Protein expression of estrogen receptors α and β and aromatase in myometrium and uterine leiomyoma. Gynecol Obstet Invest. 2012;73:113–7. 22377971 10.1159/000330700

[18] Matsuo H Kurachi O Shimomura Y Samoto T Maruo T. Molecular bases for the actions of ovarian sex steroids in the regulation of proliferation and apoptosis of human uterine leiomyoma. Oncology (Huntingt). 1999;57(Suppl 2):49–58. 10.1159/000055275 10545803

[19] Ramaiyer MS Saad E Kurt I Borahay MA. Genetic mechanisms driving uterine leiomyoma pathobiology, epidemiology, and treatment. Genes (Basel). 2024;15:558. 38790186 10.3390/genes15050558 PMC11121260

[20] Kwas K Nowakowska A Fornalczyk A. Impact of contraception on uterine fibroids. Medicina (Kaunas). 2021;57:717. 34356998 10.3390/medicina57070717 PMC8303102

[21] Hoffman SR Smith JS Funk MJ. Combined oral contraceptive utilization and uterine fibroid incidence: A prospective study in a cohort of African-American women. PLoS One. 2024;19:e0303823. 38781223 10.1371/journal.pone.0303823 PMC11115284

[22] Sangkomkamhang US Lumbiganon P Pattanittum P. Progestogens or progestogen-releasing intrauterine systems for uterine fibroids (other than preoperative medical therapy). Cochrane Database Syst Rev. 2020;11:CD008994. 33226133 10.1002/14651858.CD008994.pub3 PMC8094271

[23] Lin KY Yang CY Lam A Chang CY Lin WC. Uterine leiomyoma is associated with the risk of developing endometriosis: a nationwide cohort study involving 156,195 women. PLoS One. 2021;16:e0256772. 34437644 10.1371/journal.pone.0256772 PMC8389431

